# Stromal matrix metalloprotease-13 knockout alters Collagen I structure at the tumor-host interface and increases lung metastasis of C57BL/6 syngeneic E0771 mammary tumor cells

**DOI:** 10.1186/1471-2407-13-411

**Published:** 2013-09-05

**Authors:** Seth W Perry, Jill M Schueckler, Kathleen Burke, Giuseppe L Arcuri, Edward B Brown

**Affiliations:** 1 Department of Biomedical Engineering, University of Rochester School of Medicine and Dentistry, 601 Elmwood Avenue, Rochester, NY 14642, USA

**Keywords:** Two-photon, Microscopy, Cancer, Second harmonic generation, Collagen, SHG, Tumor, MMP, Matrix metalloprotease, MMP-13, Intravital, Imaging, In vivo, Multiphoton, Intrinsic, Fluorophore

## Abstract

**Background:**

Matrix metalloproteases and collagen are key participants in breast cancer, but their precise roles in cancer etiology and progression remain unclear. MMP13 helps regulate collagen structure and has been ascribed largely harmful roles in cancer, but some studies demonstrate that MMP13 may also protect against tumor pathology. Other studies indicate that collagen’s organizational patterns at the breast tumor-host interface influence metastatic potential. Therefore we investigated how MMP13 modulates collagen I, a principal collagen subtype in breast tissue, and affects tumor pathology and metastasis in a mouse model of breast cancer.

**Methods:**

Tumors were implanted into murine mammary tissues, and their growth analyzed in Wildtype and MMP13 KO mice. Following extraction, tumors were analyzed for collagen I levels and collagen I macro- and micro-structural properties at the tumor-host boundary using immunocytochemistry and two-photon and second harmonic generation microscopy. Lungs were analyzed for metastases counts, to correlate collagen I changes with a clinically significant functional parameter. Statistical analyses were performed by t-test, analysis of variance, or Wilcoxon-Mann–Whitney tests as appropriate.

**Results:**

We found that genetic ablation of host stromal MMP13 led to: 1. Increased mammary tumor collagen I content, 2. Marked changes in collagen I spatial organization, and 3. Altered collagen I microstructure at the tumor-host boundary, as well as 4. Increased metastasis from the primary mammary tumor to lungs.

**Conclusions:**

These results implicate host MMP13 as a key regulator of collagen I structure and metastasis in mammary tumors, thus making it an attractive potential therapeutic target by which we might alter metastatic potential, one of the chief determinants of clinical outcome in breast cancer. In addition to identifying stromal MMP13 is an important regulator of the tumor microenvironment and metastasis, these results also suggest that stromal MMP13 may protect against breast cancer pathology under some conditions, a finding with important implications for development of chemotherapies directed against matrix metalloproteases.

## Background

The structure and function of tumor extracellular matrix (ECM) play critical roles in cancer initiation and outcome [1,2]. More specifically, organization or reorganization of collagen, a key structural component of the ECM, has been shown to be an important factor in tumor genesis, progression, and/or metastasis [3-9]. Tumor cells have been shown to migrate preferentially along aligned collagen fibers [10,11], and others have reported particular “tumor associated collagen signatures” (TACS) – i.e. patterns of collagen alignment around the tumor boundary – that may be associated with breast cancer tumor invasion into host stroma [5] and patient survival [12]. However specific functional mechanisms or molecular mediators that lead to such collagen reorganizations, with consequent effects on tumor progression and metastasis, remain largely undefined and would represent attractive novel therapeutic targets for breast cancer.

Some likely mediators of collagen structure at the tumor-host interface are matrix metalloproteases (MMPs). MMPs are key regulators of the ECM and collagen remodeling [13,14], and are also frequently implicated in cancer [15]. MMP-13 (collagenase-3) was originally isolated from human breast cancer tissue [16], and has been shown to be an important contributor to breast (and other) cancer pathology [15,17,18]. As a collagenase with fairly broad substrate specificity, MMP-13 is capable of cleaving several collagen subtypes including fibrillar collagens I, II, and III [15]. These fibrillar collagens are detectable by second harmonic generation (SHG) microscopy, an optical imaging approach that is increasingly being used to provide diagnostic insights into cancer biology [9], and which has been used extensively in TACS analysis [5,12,19]. Collagen I is the most abundant fibrillar collagen in mammals [20], is a substrate for MMP-13 [15], and is typically increased in breast tumor- versus normal mammary gland-associated stroma [21,22]. Finally, Col1a1 transgenic mice with degradation resistant collagen I have been used for TACS analysis of increased collagen deposition in a mammary tumor model [19]. Therefore, in this study we sought to determine whether direct *in vivo* genetic manipulation of host MMP-13 alters collagen I organization at the mammary tumor-host boundary (i.e. TACS), with demonstrable effects on tumor metastasis.

## Methods

### Cells and reagents

Murine medullary mammary adenocarcinoma (E0771) cells syngeneic with C57BL/6 mice (Roswell Park Cancer Institute, Buffalo, NY) were grown in RPMI 1640 medium (Gibco/Invitrogen, Carlsbad, CA) supplemented with 10% gamma-irradiated defined fetal bovine serum (HyClone/Thermo-Fisher, Waltham, MA) and 100 ug/ml Primocin antibiotic (InvivoGen, San Diego, CA). For mammary tumor implantation experiments, cells were harvested in 0.25% trypsin/EDTA, centrifuged, re-suspended in sterile PBS, and kept on ice until implantation into a mammary fat pad.

### Tumor implantation

Congenic female C57BL/6 wildtype (WT) or MMP-13 knockout (MMP13 KO) mice [23] were used for E0771 tumor implantation experiments at 15–19 weeks of age. Animals were anesthetized with ketamine/xylazine (90/9 mg/kg) delivered intraperitoneally (i.p.). The animals’ ventral surfaces were depilated, followed by implantation of 1×10^5^ E0771 mammary tumor cells into the right inguinal mammary fat pads using a 27-gauge needle. Caliper-measured tumor sizes were recorded on days 12, 19, and 28 of the experiments. On Day 28 post-implantation, animals were sacrificed by i.p. sodium pentobarbital injection and subsequent cervical dislocation. The E0771 mammary tumors were excised, and immediately snap-frozen on dry ice for subsequent cryo-sectioning and immunohistochemistry. Lungs were excised, fixed in 10% neutral-buffered formalin, then paraffin embedded, sectioned, and hematoxylin-eosin (H&E) stained for analysis of lung metastases. Procedures were performed in accordance with the University Committee on Animal Resources (UCAR).

### Immunohistochemistry

Snap-frozen tumors were cryo-sectioned (−21°C) at 20 um, then static-mounted on positively charged slides. For immunohistochemistry (IHC), sections were cold-fixed (−20°C) for 20 minutes in 3:1 acetone/methanol, rehydrated 2 × 5 minutes in sterile PBS, then blocked for one hour (5% BSA, .2% Triton-X 100 in PBS). Primary antibody for Collagen I (Abcam #21286, Cambridge, MA) was then applied for 2 hours at room temperature in a humidified chamber, diluted 1:200 in 0.5% BSA, .2% Triton-X 100 in PBS, followed by 2 × 5 minutes PBS wash, then two hours of Alexa Fluor 594-conjugated goat anti-rabbit IgG (1:500 in the same diluent as the primary; Invitrogen, Carlsbad, CA). Optimal antibody dilutions and incubation times were pre-determined empirically. Following staining for Collagen I protein, tumor sections were washed and mounted in ProLong Gold Antifade reagent (without DAPI; Invitrogen, Carlsbad, CA), then allowed to dry for 24 hours before imaging. Similar procedures were used for IHC against MMP-13 (Millipore #ab8120, Billerica, MA).

### Imaging and image analysis

Slides were imaged by a blinded observer using a custom-built multi-photon microscope. A Mai Tai Titanium:sapphire laser (Newport/Spectra Physics, Santa Clara, CA) provided two-photon (2P) excitation (100 fs pulses at 80 MHz and 810 nm) for simultaneously epidetecting backwards-directed SHG (B_SHG_) and immunofluorescence (IF) signals from Collagen I fibers in the excised mammary tumors. Beam scanning and image acquisition were performed with a custom-modified Fluoview FV300 confocal scanner interfaced with a BX61WI upright microscope (Olympus, Center Valley, PA), with an Olympus XLUMPLFL20xW water immersion lens (20×, 0.95 N.A.). Backscattered SHG (HQ405/30m-2P emission filter, Chroma, Rockingham, VT; HC125-02 PMT, Hamamatsu Corporation, Hamamatsu, Japan) and 2P-excited Collagen I IF (Chroma HQ635/30 m-2P emission filter; HC125-01 Hamamatsu PMT) signals were separated from the 810 nm excitation beam by a short pass dichroic mirror (Chroma 670 DCSX), and simultaneously captured in two distinct channels (using a 475 DCSX Chroma long pass dichroic, and the emission filters and PMTs above) on every scan. Resulting two-channel (B_SHG_ and IF) images are 680 microns across. Laser power was monitored and kept constant throughout each experiment and across experimental repetitions, as were PMT voltage, gain, and offset.

Because MMP13 was knocked out from the host animal, for the results described herein we analyzed images representing random tumor-host interface regions, i.e. random “outer edge” regions of tumors. Images from these areas were obtained as z-stacks (1 um step size) taken over the entire 20 um thickness of the tissue section. For each channel (B_SHG_ and collagen I IF), maximum projection images were taken from each stack, then image analysis was performed with ImageJ as follows. For each slide (usually 2–3 tumor sections/slide) and for each channel, background was defined by the average pixel counts of a laser-excited image taken from an area of the slide with no tissue, and subtracted from the raw B_SHG_ and IF images. These background corrected images were used for image analysis in ImageJ. For assessment of collagen I protein levels, mean pixel intensity of collagen I IF was measured over the tumor periphery FOVs. For coherency analysis, the coherency parameter (see below, and [24-26]) was calculated on these same collagen I IF images using the ImageJ plugin OrientationJ (open source, written by Daniel Sage, Biomedical Image Group, EPFL, Switzerland; http://bigwww.epfl.ch/demo/orientation/). This coherency parameter was quantified on a pixel-by-pixel basis, then averaged over all pixels in an image to produce a single coherency value for each image, which could then be averaged across all images in each experimental group. For normalized SHG calculations (i.e. B_SHG_ normalized to collagen I IF), the B_SHG_ and collagen I IF images were thresholded to select for collagen I-positive features and to reduce artifactual effects from non-specific background in either channel, applying the same thresholding standard to images from all experimental groups. The B_SHG_ channel was then “masked” to the thresholded collagen I IF channel, so that only SHG pixels that *were also positive for collagen I immunofluorescence* were analyzed. Dividing the mean B_SHG_ pixel intensity from these masked B_SHG_ images, by the mean IF pixel intensity from the corresponding Collagen I IF images from the same FOV, produces a ratiometric value which represents B_SHG_ normalized to Collagen I protein levels on a pixel-by-pixel basis over the exact same XYZ pixel space, for each tumor periphery image. This method of analysis allows us to analyze only the B_SHG_ signal that is primarily restricted to collagen I, and provides a “normalized” B_SHG_ value for collagen I. Since SHG is sensitive to both collagen microstructural properties and collagen abundance [9,27], this “normalized” B_SHG_ parameter allows us to primarily assess changes in collagen I microstructural properties with reduced sensitivity to changes in collagen I expression levels, and similar strategies have been employed by others previously [28,29]. To calculate the forward SHG (F_SHG_) to B_SHG_ ratio (F_SHG_/B_SHG_), both F_SHG_ and B_SHG_ were captured simultaneously above (B_SHG_) and below (F_SHG_) the slide specimen in two channels using the microscope setup previously described [30]. F_SHG_ and B_SHG_ images were simultaneously collected as stacks of 11 images spaced 3μm apart, within a 660 μm field of view. Four images were taken from each tumor sample around the tumor boundary at the tumor-host interface, with 2 tumor samples analyzed per animal from the same cohorts of WT and MMP13 KO animals. Image analysis was conducted with ImageJ. Each stack was maximum intensity projected, serving as an “autofocus” for the effectively single layer of collagen. Projected images were background subtracted using a maximum intensity projection of a matching 11 image scan taken with a closed shutter, then background subtracted F_SHG_ and B_SHG_ images were divided to create an F_SHG_/B_SHG_ ratio image. For each image a common threshold was applied to all images to distinguish collagen pixels from background pixels and to select for fibers likely to be collagen I, and subthreshold background (i.e. non collagen fiber) pixels were excluded from analysis by binary masking. The average pixel value these non-background collagen fiber pixels was calculated from all F_SHG_/B_SHG_ ratio images in the same cohort of MMP13 KO and WT animals, and expressed as mean F_SHG_/B_SHG_ ± SEM.

### Evaluation of lung metastases

For evaluation of lung metastases, lungs were excised from tumor bearing experimental animals as described above, fixed in 10% neutral-buffered formalin, then paraffin embedded. Five-micron rotary microtome sections were taken through both lobes of the lung, mounted on positively charged slides, then H&E stained. H&E-stained lung sections were evaluated for lung metastases by a trained blinded observer using brightfield microscopy (Olympus BX-51, Center Valley, PA). Metastatic infiltrating tumor cells were identified by several criteria including: high ratio of hematoxylin relative to eosin, surrounding abnormalities in lung structure, abnormal shape/size of nuclei and/or presence of abnormal mitotic spindles, and differences in cell shape and size, with ≥ 1 infiltrating tumor cells counted as a metastatic event. Results are presented as mean number of lung metastases/cm^2^ ± SEM for each treatment group.

### Statistical analysis

Statistical analysis was performed using Kaleidagraph (Synergy Software, Reading, PA) or Prism 5 (GraphPad Software, La Jolla, CA) software. Student’s t-tests (unpaired), ANOVA with protected Fisher LSD post-tests for planned comparisons, or Wilcoxon-Mann–Whitney tests were used for statistical analyses as appropriate. p-values ≤ .05 were considered significant.

## Results

### Stromal MMP13 knockout increases mammary tumor Collagen I content, but decreases Collagen I ordering, at the tumor-host interface

**Figure 1:**
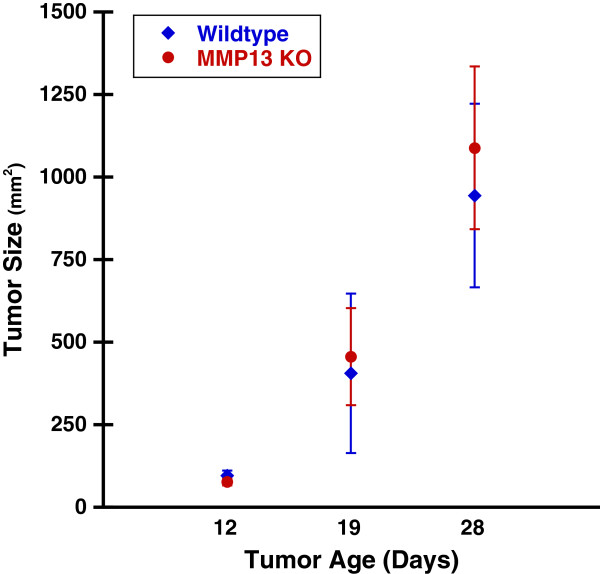
**Host MMP13 knockout does not alter mammary tumor size.** E0771 mouse mammary tumor cells were implanted into the mammary fat pad of congenic female C57BL/6 WT or MMP13 KO mice as described in Methods. Tumors were measured with calipers at days 12, 19, and 28 post-implantation, and tumor volume calculated. There was no difference in tumor volume between WT and MMP13 KO mice at any of the time points, indicating that MMP13 knockout does not alter E0771 mouse mammary tumor size. Plot represents mean tumor volumes ± SEM from a cohort of WT (blue) (n=6) and MMP13 KO (red) (n=4) mice.

**Figure 2:**
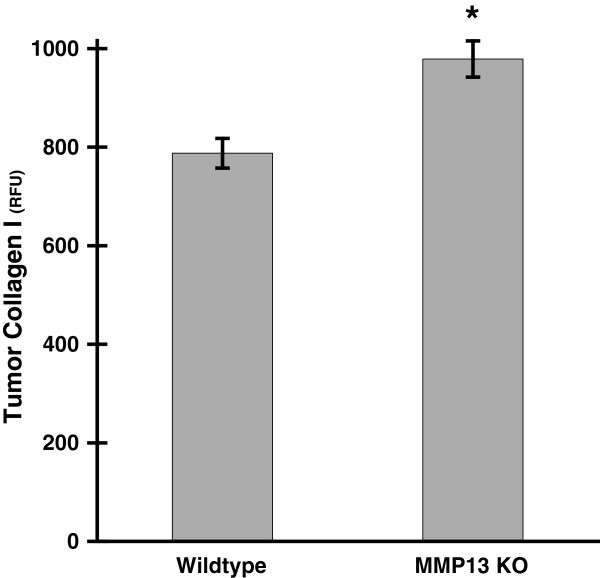
**MMP13 knockout increases Collagen I levels in E0771 mammary tumors.** WT and MMP13 KO mice were implanted with E0771 mammary tumors as described. Following excision of the primary tumor, tumor boundary regions were assessed for Collagen I levels by immunochemistry. Immunofluorescence signal was captured by two-photon excitation microscropy of fields of view (FOV) from random tumor boundary regions. Z-stacks from each FOV were maximum intensity projected and background subtracted, and fluorescent intensities from the resultant images were quantified with ImageJ and then expressed as mean anti-Collagen I immunofluorescence ± SEM from n=16 (WT) and n=14 (MMP13 KO) tumor FOVs from the same cohort of animals. Collagen I levels in tumor peripheries were significantly increased in MMP13 KO versus WT mice (*p < .0004).

**Figure 3:**
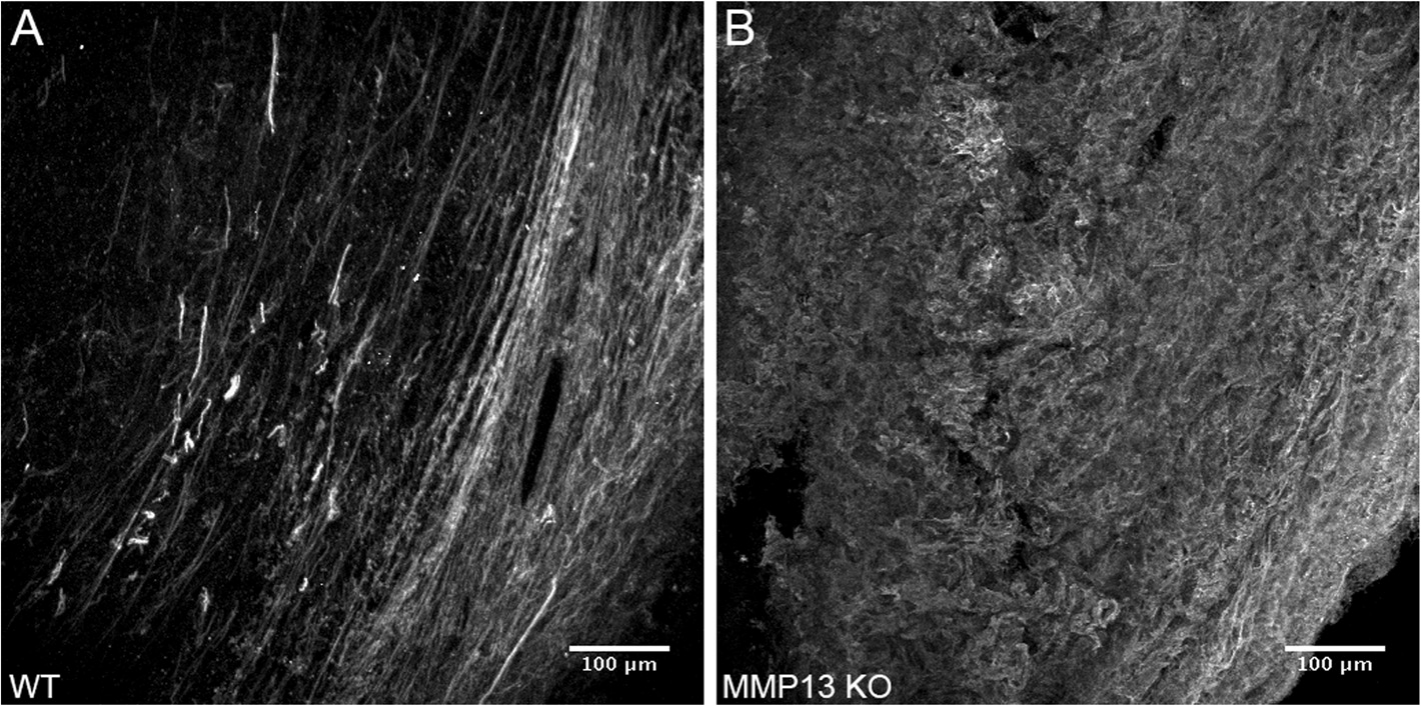
**MMP13 knockout decreases Collagen I ordering at E0771 mammary tumor boundaries.** WT and MMP13 KO mice were implanted with E0771 mammary tumors as described. Following excision and sectioning of the primary tumor, tumor boundary regions were assessed for Collagen I spatial organization by qualitative (this figure) and quantitative (next figure) analysis of anti-Collagen I immunofluorescence signal. Images of Collagen I signal were taken as described in Figure 2. Note that WT mice **(Panel A)** exhibited a much more organized and ordered Collagen I structure, characterized in particular by longer and thicker Collagen I fibers oriented more parallel to the tumor boundary, compared to MMP13 KO mice **(Panel B)** which demonstrated a more diffuse Collagen I pattern with fewer pronounced, rod-like Collagen I fibers.

Murine medullary breast adenocarinoma (E0771) tumor cells were implanted into mouse mammary fat pads for 28 days. Consistent with other reports [31], MMP13 KO did not alter mammary tumor size in our E0771 mammary tumor model (Figure 1). MMP-13 expression was decreased in tumors from the MMP13 KO mice versus WT (Additional file 1: Figure S1A) and moreover, in the WT but not MMP13 KO mice, MMP13+ cell bodies were found around the tumor periphery which suggested the presence of peritumor (and possibly infiltrating) MMP13+ stromal cells in WT but not MMP13 KO mice (compare Additional file 1: Figures S1B and S1C, respectively). Also at the tumor periphery, Collagen I protein levels were significantly increased in MMP13 KO versus wildtype animals as quantified by immunofluorescence staining (Figure 2), suggesting that depletion of this collagen degrading enzyme from host cells increases tumor Collagen I content. However, despite this increase in Collagen I content at the tumor boundaries in the MMP13 KO mice, overall collagen *ordering* at tumor boundaries was decreased (i.e. more random) in these MMP13 KO mice versus WT controls. Figure 3 highlights the decreased ordering in MMP13 KO mice, with WT mice demonstrating many robust Collagen I fibers more frequently oriented parallel to the tumor boundary, closely resembling a TACS-2 arrangement as previously described [5,12,19] (Figure 3A). Compared to these WT mice, MMP13 KO mice in contrast, although they had *greater* overall collagen I content (Figure 1), displayed fewer robust linear collagen I fibers, and a more randomly oriented collagen I distribution closely resembling a TACS-1 arrangement (Figure 3B). In adjacent sections of the same WT tumor as shown in Figure 3A, antibody labeling for MMP13 around the tumor periphery appeared to be closely localized with the robust TACS-2 patterned Collagen I fibers oriented parallel to the tumor boundary (Additional file 2: Figure S2), thus further implicating MMP-13 as likely to be a key orchestrator of these observed TACS changes in Collagen I organization.

To quantify these differences in local orientations of collagen I fibers, we performed tensor analysis of local image structure to calculate the coherency parameter (C), which is the ratio of the difference and sum of the largest (λ_max_) and smallest (λ_min_) tensor eigenvalues (averaged for all pixels over the image FOV), as follows:

(1) $$C=\left(\lambda_{\max}-\lambda_{\min}\right)/\left(\lambda_{\max}+\lambda_{\min}\right)$$

**Figure 4:**
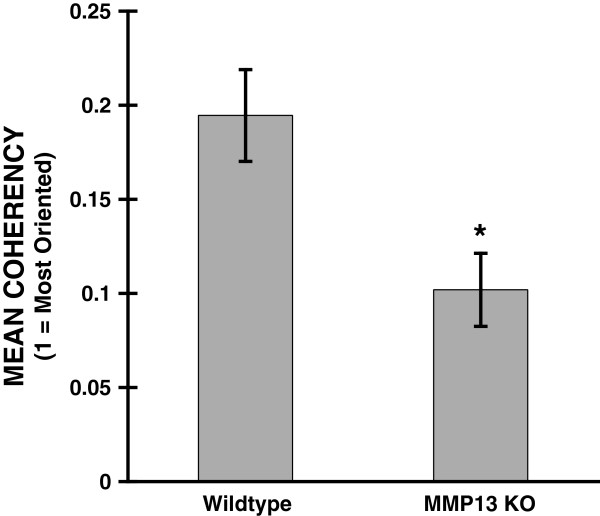
**Quantifying decreased Collagen I ordering at the E0771 mammary tumor boundary in MMP13 KO mice.** WT and MMP13 KO mice were implanted with E0771 mammary tumors as described, and images of anti-Collagen I immunofluorescence staining at the tumor-host interface were obtained as described in Figure 3. As described in Methods and Results, to quantitatively assess Collagen I ordering we calculated the mean coherency parameter averaged over all pixels in each image, thus producing a single coherency value for each image. This coherency value for each image was averaged for n=16 (WT) and n=14 (MMP13 KO) tumor boundary FOV images (± SEM) from the same cohort of animals to produce this plot. Mean coherency was significantly decreased in the MMP13 KO versus WT mice (*p < .007), reflecting less organized (more randomly oriented) collagen I structure. Coherency values for each image were produced using OrientationJ (http://bigwww.epfl.ch/demo/orientation/), then graphed in Kaleidagraph (Synergy Software, Reading, PA).

**Figure 5:**
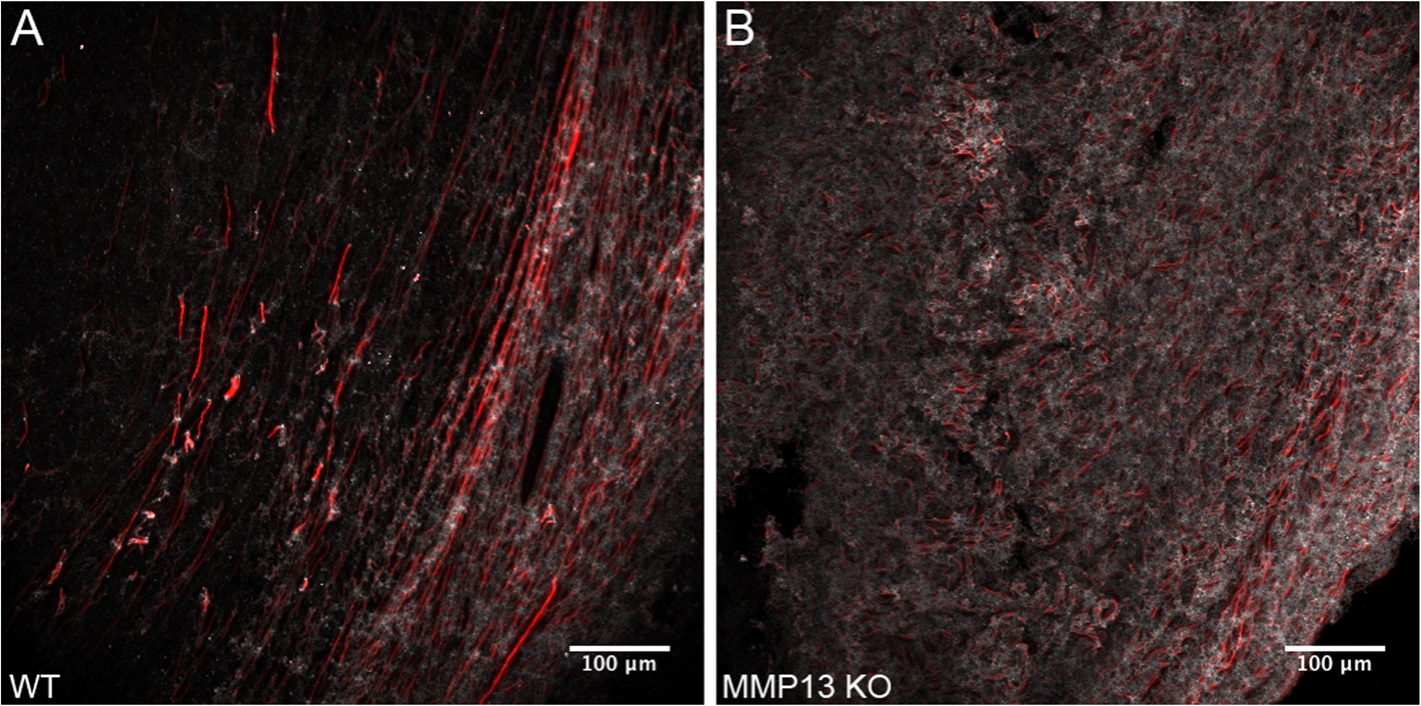
**Coherency maps of decreased Collagen I ordering at the E0771 mammary tumor boundary in MMP13 KO mice.** The same representative images as in Figure 3 were combined with their respective pixel-by-pixel coherency maps (calculated as described for Figure 4) as Hue-Saturation-Brightness (HSB) images (H = Constant; S = Coherency; B = Original Image), such that increased amounts of “bright red” signal reflects greater coherency. Compare more bright red signal signifying greater coherency in WT **(Panel A)**, versus less bright red signal and lesser coherency in MMP13 KO **(Panel B)**. Coherency maps were produced using Orientation J (http://bigwww.epfl.ch/demo/orientation/).

With the upper bound C = 1 indicating highly oriented structures, and the lower bound C = 0 indicating high isotropy [24-26]. This analysis clearly indicated that Collagen I in mammary tumor peripheries of WT mice was more highly oriented (i.e. more ordered; C closer to 1), than in the mammary tumor peripheries of MMP13 KO mice (less ordered, C closer to 0) (Figure 4). Figure 5 shows a pseudo-colored map of these coherency values using the same images as in Figure 3 (more visible bright red areas = higher coherency).

Together these results suggest that depletion of host (stromal) MMP13 – a key collagen degrading enzyme – increased total collagen I content, but reduced collagen I organization, in the periphery of E0771 mammary tumors grown in MMP13 KO versus WT mice.

### Stromal MMP13 knockout alters Collagen I microstructure at the tumor-host interface

SHG results when two photons (e.g. as provided by the near-IR titanium sapphire laser in a multiphoton microscope), interacting simultaneously with a non-centrosymmetric target such as collagen fibers, combine to produce a new photon with exactly twice the energy and half the wavelength of the interacting photons [9,32-36]. As a coherent phenomenon, SHG is intrinsically sensitive to spacing and regularity of scatterers, and hence can be utilized to detect changes in several aspects of collagen *micro*structure including regularity of the arrangement of collagen fibrils within larger collagen fibers; interfibril spacing; and fibril diameter, tilt angle, or pitch angle [4,7,9,27,34,37-41]. Collagen I is a particularly strong SHG emitter *in vivo*[7], is a substrate for MMP13 [15], is increased in breast cancer stroma [21,22], and is an important contributor to TACS [19]. Figures 2, 3, 4 and 5 assessed collagen I *macro*structural properties (i.e. gross fiber orientations and arrangement) at the tumor-host interface, and to further these findings, we now wished to analyze collagen I *micro*structural properties at the tumor-host interface as assessed by SHG. For these reasons, we restricted our SHG analysis to the intensity of SHG signals emanating primarily from collagen I fibers, specifically using the “normalized B_SHG_” as described in Methods to provide an SHG measure of collagen I *micro*structural properties independent of changes in collagen I protein levels, as well as to gain particular insight into changes in the effective diameter or packing arrangement/density of fibrils within the SHG focal volume [4,27,30,34,42].

Figures 3, 4 and 5 demonstrated that while tumors in both WT and MMP13 KO mice contained both “diffuse” and “large fiber” patterns of collagen I, there was proportionately more “diffuse” collagen I with apparently thinner fiber structure or bundling on average in MMP13 KO tumors, versus proportionately more “large fiber” collagen I in WT tumors (often ordered parallel to the tumor boundary resembling a TACS-2 signature, e.g. Figure 3A). Therefore we hypothesized that knockout of MMP13 collagenase activity could cause differences in collagen I *micro-*structural properties – e.g. regularity or density of collagen fibrils within larger collagen fibers; fibril spacing; and fibril diameter, tilt angle, or pitch angle [4,7,9,27,34,37-41] – which might in turn account for the different collagen I *macro*structural phenotypes observed in MMP13 KO versus WT mice.

**Figure 6:**
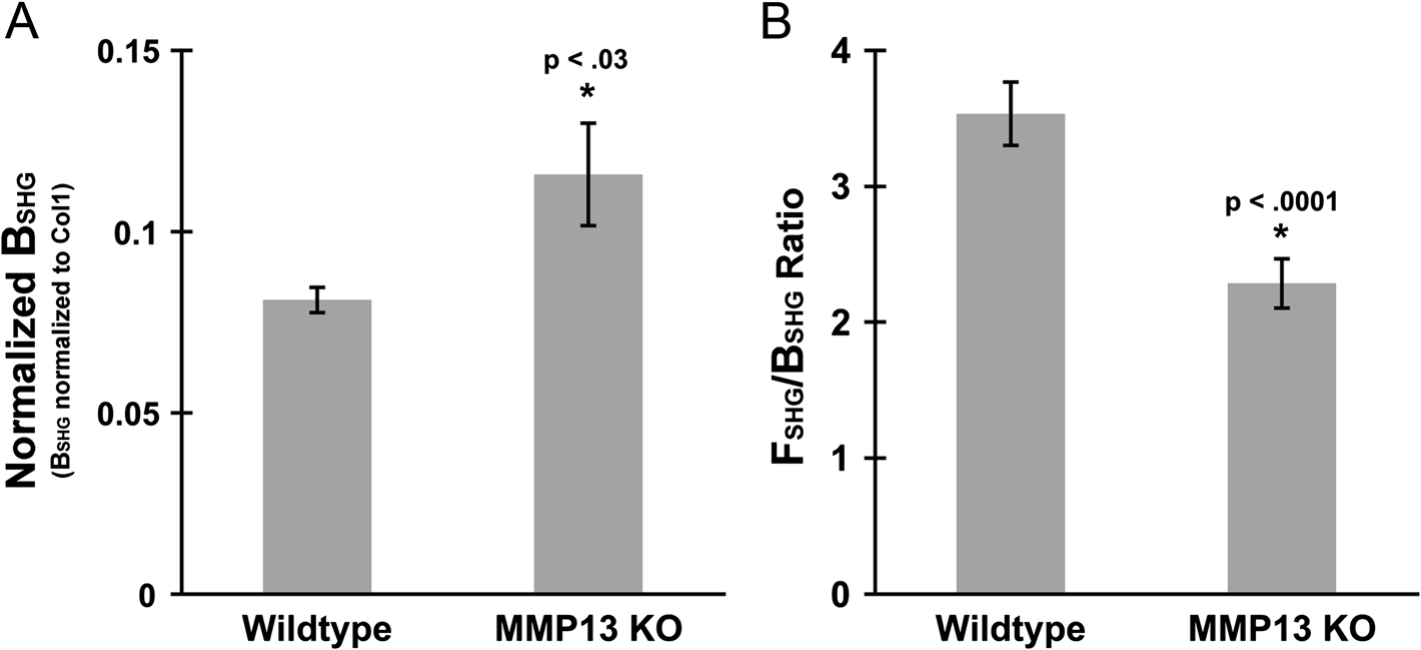
**MMP13 knockout alters Collagen I microstructure at the tumor-host interface.** WT and MMP13 KO mice were implanted with E0771 mammary tumors. **(A)** Following excision of the primary tumor, tumor boundary regions were labeled with anti-collagen I. Collagen I immunofluorescence and B_SHG_ signal were captured simultaneously in separate epidetection channels by two-photon excitation microscopy of fields of view (FOV) from random tumor boundary regions. Z-stacks from each FOV were maximum intensity projected and background subtracted, SHG and collagen I immunofluorescence signals masked to the same pixel areas, then from these masked images “normalized B_SHG_” (i.e. B_SHG_ normalized to collagen I levels) was calculated as mean B_SHG_ pixel value/mean collagen I immunofluorescence pixel value ± SEM for all images over the same XYZ pixels. This ratiometric normalized collagen I SHG value was calculated for 16 (WT) and 14 (MMP13 KO) tumor FOVs from the same cohort of animals, and was higher in MMP13 KO versus WT tumor boundaries (p < .03). **(B)** From the same cohort of animals, we also calculated F_SHG_/B_SHG_ values, which provides insight into microstructural collagen changes, specifically the sub-resolution diameter and/or packing density/arrangement of collagen fibrils. F_SHG_/B_SHG_ was significantly decreased in tumor boundary regions of MMP13 KO versus WT mice (p < .01).

As described here and in Methods, we measured collagen I-normalized B_SHG_ in the same WT and MMP13 KO animals and tumor-host interface regions as depicted in Figures 1, 2, 3, 4 and 5. We found that normalized B_SHG_ was significantly higher in the E0771 tumor peripheries of MMP13 KO versus WT mice (Figure 6A), suggesting different collagen I microstructure between these two groups [28,29]. To validate and complement these findings, we also measured the F_SHG_/B_SHG_ ratio in these same groups and tumor-host interface regions. SHG is emitted both forwards (away from the incoming laser) and backwards (back towards the incoming laser, i.e. epi-directed) from the SHG-generating scatterers in the focal volume, and the F_SHG_/B_SHG_ ratio is an additional SHG parameter that is primarily sensitive to the spatial extent of SHG-generating scatterers along the optical axis, i.e. the effective diameter or packing arrangement/density of collagen fibrils within the SHG focal volume [4,27,30,34,42]. We found this F_SHG_/B_SHG_ ratio was significantly decreased in the E0771 tumor peripheries of MMP13 KO versus WT mice (Figure 6B). Together these two pieces of data suggest collagen I *micro*structure is altered in MMP13 KO versus WT mice, which may in turn relate to the observed changes in collagen I *macro*structure (e.g. differences in average fiber density, apparent thickness, and organization) seen in Figures 3, 4 and 5.

Together these results suggest that stromal host MMP13 depletion alters both collagen I *macro*structural (i.e. fiber arrangement, ordering, and orientation; Figures 2, 3, 4 and 5), and molecular (fibril) *micro*structural properties (as quantified by collagen I normalized B_SHG_ and F_SHG_/B_SHG_; Figure 6) at the tumor periphery. Since WT tumor peripheries showed significantly more robust “rod like” collagen I fibers (often in a more TACS-2-like orientation), compared to the higher proportion of “diffuse” fibers in MMP13 KO animals (often in a more TACS-1-like orientation) (Figure 3), these results further suggest the possibility that MMP13 KO changes collagen I microstructure in ways that 1. Could alter collagen’s ability to form and orient larger, more rod-like fibers, and/or 2. May shift the relative balance between “diffuse” and “rod-like” collagen I phenotypes.

### Stromal MMP13 knockout increases mammary tumor metastasis to lung

Collagen is a key component of the extracellular matrix (ECM) which regulates cell behavior and motility [1,8,10,43], metastasizing breast tumor cells in particular have shown a propensity to “escape” the tumor by traveling along collagen fibers [5], and particular collagen patterns or “TACS” at the breast tumor periphery are associated with poor survival [12]. Therefore the observed collagen I macro- and micro-structural changes at the tumor-host interface might be expected to affect tumor metastasis.

**Figure 7:**
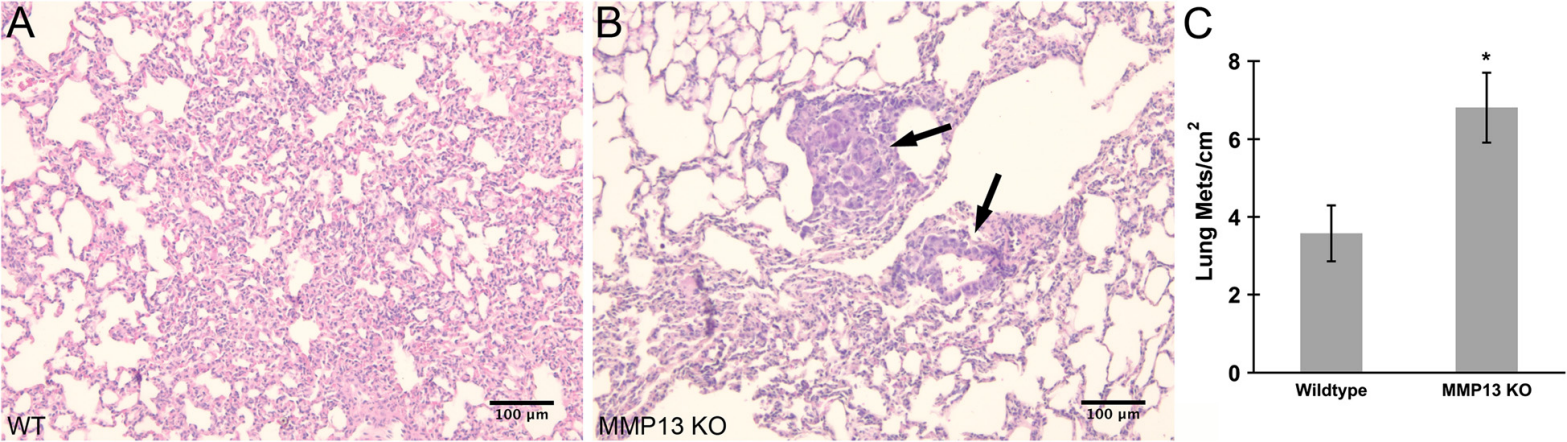
**MMP13 knockout increases mammary tumor metastasis to lung.** E0771 mouse mammary tumor cells were implanted into the mammary fat pad of congenic female C57BL/6 WT or MMP13 KO mice as described in Methods. At day 28 post-implantation, lungs were excised and processed for H&E staining for analysis of metastases. Representative images from the **(A)** WT and **(B)** MMP13 KO animals show increased metastastic burden (see arrows indicating metastases) in the MMP13 KO group. **(C)** Metastases/cm^2^ were counted and averaged from 10 equidistant sampling step sections through both lobes of the lung per animal from a cohort of WT (n=6) and MMP13 KO (n=4) mice, demonstrating that MMP13 KO mice had nearly double the number of lung metastases compared to their WT counterparts (*p < .006).

For these reasons, and because breast tumor metastasis to lung is associated with poor prognosis [44], we wished to determine whether the changes in collagen I macro- and micro-structural properties demonstrated above could be associated with changes in this clinically significant parameter. Indeed, we found that along with their differing collagen I macro- and micro-structural properties (Figures 2, 3, 4, 5 and 6), our MMP13 KO animals also had roughly double the number lung metastases compared to WT animals (Figure 7). This difference in metastasis was not due simply to differences in tumor burden between the WT and MMP13 KO animals, as tumor burden was unchanged (Figure 1).

## Discussion

The ECM, and collagen in particular, are increasingly believed to play important roles in cancer etiogenesis, progression, and outcome [1-9]. Several previous reports have demonstrated that tumor cells may preferentially travel along collagen fibers [10,11], which may represent an important pathway by which invading cells metastasize [5,11]. Accordingly, collagen fibers oriented perpendicularly to the tumor boundary in what has been termed a “TACS-3” configuration, have been associated with increased invasiveness into host stroma [5] and with decreased patient survival [9,12]. In contrast, TACS-2 collagen configuration- i.e. straight “taut” fibers often parallel to the tumor boundary-was associated with regions of decreased tumor invasiveness [5].

MMPs have been implicated in many cancers including breast cancer, most likely due to their ability to modulate this collagen- and ECM-rich extracellular environment [45]. While a majority of studies have found pro-tumorigenic roles for most MMPs, a growing body of literature suggests that some or even many MMPs may have anti-cancer effects as well [46,47]. Protective effects of MMPs against tumor pathology may in part account for the relative failure of MMP inhibitors as effective chemotherapeutics [46,48,49], and this concept is further supported by evidence that endogenous tissue inhibitors of matrix metalloproteinases (TIMPs) can themselves be cancer-promoters [50-53]. MMP13 in particular has widely been found to promote cancer pathology, but a few emerging reports including this one find an apparently protective role for MMP13 in cancer and other diseases under some conditions [54,55]. Moreover, there remains limited understanding of exactly how MMP-13 interactions with collagen impact tumor pathology – for example, what structural changes result from these interactions, and how do these changes promote or protect against tumor pathology? In the studies described here, we have provided further insights into these important questions.

Herein we extended this previous work by demonstrating that *in vivo* genetic ablation of host MMP-13 in a mouse tumor model leads to altered collagen I macro- and micro-structure at the tumor-host interface, and increases mammary tumor metastasis to the lungs, a clinically significant functional outcome measure. This represents a direct experimental manipulation of the TACS stage of the tumor and therefore implicates stromal MMP13 as one driver of TACS evolution in breast tumors. These results are important because they help clarify the role of host MMP13 in tumor collagen dynamics, breast cancer pathogenesis, and metastasis. They are also important because this is one of few studies that have found a potentially protective effect for host MMP13 in the context of cancer pathology, and it is important to understand these intricacies of MMP13’s roles in cancer biology – i.e. when, where, and how it may have protective versus deleterious functions in cancer – in order to develop effective, targeted MMP-based therapies that do more good than harm.

Due to the fact that tumor cells migrate preferentially along aligned collagen fibers [10,11], our findings of decreased metastasis (Figure 7) associated with collagen I TACS-2 patterning (i.e. fibers parallel to the tumor boundary: Figure 3A) in WT mice, taken with earlier studies demonstrating decreased tumor invasiveness also in TACS-2 areas [5], together support the *hypothesis that alignment of collagen fibers parallel to the tumor boundary may effectively serve as a literal “barrier” or “diversion” to metastasizing tumor cells.* Continuing this argument, it is easily seen how the contrasting TACS-3 pattern found in the literature, i.e. collagen fibers oriented perpendicular to the tumor boundary, may allow metastasizing cells to travel outward along these collagen “tracks” to more readily invade the host [5,10]. While we did not find significant TACS-3 patterning in our model system, our data suggest that the TACS-1 patterning (i.e. increased, more diffuse collagen deposition) resulting from knockout of stromal MMP13 *may also* result in increased metastasis if present in late-stage tumors, we posit because it is far less “barrier like” than TACS-2 patterning (e.g. compare Figures 3A and 3B, respectively), thus allowing for relatively easier escape of metastasizing tumor cells, possibly due to less diversion of those cells onto paths parallel to the tumor boundary.

In these previous reports, TACS-1 patterns were not investigated in detail for metastases effects, because in their model the TACS-1 collagen pattern tended to occur early in tumor formation before significant metastases occurred [5]. TACS-1 is characterized by dense, more diffuse collagen areas [5], consistent with the increased collagen I seen in the TACS-1 MMP13 KO group (Figure 2), which likely results from the absence of this key collagen I-degrading collagenase. Moreover, while E0771 tumor cells appear to have some level of MMP13 expression (Additional file 1: Figure S1 and unpublished data), as is common in mammary tumor cells [56-58], we believe most significant for our findings are the more strongly MMP13+ peritumor cell bodies seen in MMP13 expressing WT animals, but not in the MMP13 KO mice (compare Additional file 1: Figures S1B and S1C respectively, and unpublished data). These MMP13+ cell bodies found around the tumor periphery in WT but not MMP13 KO mice suggest the presence of peritumor (and possibly infiltrating) MMP13+ stromal cells which may contribute to the altered TACS patterns and metastases observed in WT versus MMP13 KO mice. These results, together with our finding that MMP13+ staining appears localized with the “barrier-like” Collagen I fibers around the tumor periphery in the TACS-2 (WT) condition (Additional file 2: Figure S2), all suggest that MMP13 in particular may be a principal contributor to TACS phenotype. The fact that MMP13 KO results in TACS-1 collagen patterning similar to what others have seen in “early stage” tumors in their mammary tumor models [5] suggests that the lack of MMP13 prevents the tumor stroma from progressing to a “late stage” structure. This is supported by the literature observation that MMP13 can be a critical mediator of “early stage” tumor events [59].

In addition to these *macro*structural collagen I changes, which we posit may impact tumor cells’ invasive potential, we also found changes in collagen I *micro*structure as measured by SHG. As described above and previously, SHG is sensitive to changes in collagen *micro*structure including regularity of collagen fibrils within larger collagen fibers; fibril compaction; and fibril diameter, tilt angle, or pitch angle [4,7,9,27,34,37-41], and therefore SHG signal normalized to collagen I levels can provide a quantitative measure of collagen I *micro*structure which is less dependent on changes in collagen I protein levels. Furthermore, typically as the diameter of collagen fibrils or small fibers (i.e. small bundles of fibrils) increases, their SHG becomes more forward-directed, and thus the F_SHG_/B_SHG_ ratio also increases [42]. Therefore our findings of lower collagen I B_SHG_ (Figure 6A), and higher F_SHG_/B_SHG_ (Figure 6B), in WT versus MMP KO mice is seemingly consistent with our observations of generally more large rod-like collagen I fibers in WT mice, versus apparently thinner and more diffuse collagen I fibers on average in MMP13 KO mice (Figures 3, 4 and 5). These findings also introduce the possibility that changes in collagen I *micro*structure (as measured by SHG) may alter collagen I’s ability to form larger rod-like fibers, thus altering the relative proportions of “diffuse” versus “large fiber” collagen I (and TACS patterning) in WT versus MMP13 KO mice (Figures 3, 4 and 5).

In further support of our work here, another report using mouse mammary tumor virus polyoma middle T (MMTV-PyMT) mice crossed with MMP13 KO mice, noted proportionately more “thin collagen fibers” (relative to total collagen) in tumors from MMP13 KO compared to WT mice as assessed by picrosirius red staining under linearly polarized light [31], although in this study additional macro- or micro-structural collagen changes, and collagen I in particular, were not investigated. Our data here suggest further that collagen I may be a principal contributor to these MMP13-regulated changes in collagen architecture and organization, at least in some mammary tumor models.

This hypothesis that changes in collagen I *micro*structural properties (measurable by SHG) may in turn contribute to observable changes in collagen I *macro*structure requires further investigation in future studies beyond the scope of this report, but we can propose at least several ways by which this might occur. Regulation of collagen I fibril formation, fiber length and thickness, and organization is exceedingly complex and may involve numerous cellular and biochemical interactions with collagen I, just a few of which include protease activity by many MMPs, proteoglycan interactions, and/or interactions of collagen I with other fibrillar or fibrillar-associated collagen subtypes or other ECM molecules [20,60,61]. Notably, there are several mechanisms by which MMP-13 in particular could induce collagen I *micro*structural changes, which further manifest themselves as *macro*structural changes in collagen I fiber diameter. First, MMP-13 cleaves the collagen I amino terminal non-helical telopeptide end [62], which in turn promotes lateral fiber growth whereas leaving this site intact decreases lateral growth and is associated with initial formation of thin fibrils [63,64] – thus providing robust support for our results that WT mice (i.e. with normal MMP-13 cleavage of this site) show thicker fibers, whereas MMP13-KO mice (lacking MMP-13 cleavage of this site) have proportionately more thin (diffuse) fibers. Moreover, MMP-13 has been shown to degrade decorin [65], a proteoglycan known to be a key regulator of collagen I fiber diameter [66]. Further supporting our results, less decorin (i.e. more MMP-13) has typically been associated with thicker collagen I fibers [67,68], as we saw in the WT mice. Finally, MMP-13 can degrade collagen III which may result in altered fiber-diameter regulating interactions between collagen I and collagen III [69], or between collagen I and amino-terminal propeptide of type III procollagen which is thought to interact with Collagen I to regulate fiber diameter [70,71].

## Conclusions

In this work we have directly shifted a mouse model of breast cancer from one TACS state to another by knockout of stromal MMP13, implicating stromal MMP13 as one driver of TACS state. This also altered the metastatic output in a manner consistent with the TACS literature, although the relationship between TACS and metastatic output is not necessarily causal based upon our data. This suggests that pharmacological manipulation of MMP13 activity is an attractive avenue of exploration in order to manipulate TACS state and hence attempt to alter metastatic output. In total, these novel findings that MMP13 may have beneficial roles in cancer biology by significantly altering collagen I dynamics and metastatic potential, help to further clarify MMP13’s potentially protective roles in tumor pathology and thus facilitate future design of more specifically targeted and effective MMP-based therapies that minimize risks to the patient.

## Competing interests

The authors declare that they have no competing interests.

## Authors’ contributions

SWP conceived and designed studies, acquired and analyzed data, and wrote the manuscript. JMS, KB, and GLA acquired and analyzed data. EBB conceived and designed studies and wrote the manuscript. All authors read and approved the final manuscript.

## Pre-publication history

The pre-publication history for this paper can be accessed here:

http://www.biomedcentral.com/1471-2407/13/411/prepub

## Supplementary Material

Additional file 1: Figure S1 Decreased MMP13 expression in MMP13 KO tumors. WT and MMP13 KO mice were implanted with E0771 mammary tumors as described. **(A)** Following excision of the primary tumor, MMP13 gene expression was assessed by quantitative PCR (qPCR) and normalized to 1, showing decreased MMP13 expression in MMP13 KO versus WT tumors (p < .03) in the same cohort of WT (n=6) and MMP13 KO (n=4) mice. In addition, following immunofluorescence labeling for MMP13, the tumors from the **(B)** WT mice had peritumor MMP13+ cell bodies which were not apparent in the tumors from the **(C)** MMP13 KO mice. To assure details are visible for illustrative purposes, the original grayscale MMP13 immunofluorescence is shown with “Green” LUT applied in ImageJ, with levels (screen stretch) linear and set the same for both images. Click here for file

Additional file 2: Figure S2 MMP13 at the tumor periphery is patterned like Collagen I in TACS-2. MMP13 immunofluorescence of adjacent sections of the same WT tumor as depicted in Figures 3A and 5A illustrates that MMP13 protein expression appears to align with the TACS-2 patterned Collagen I fibers depicted in Figures 3A and 5A, which are robust and oriented in a “barrier like” fashion around the tumor periphery, as is the MMP13 labeling shown here. Fiber banding patterns are visible amidst the MMP13 fluorescence labeling in this image. For illustrative purposes, the original grayscale MMP13 immunofluorescence is shown with a spectral lookup table (“Fire” LUT in ImageJ) applied and linear screen stretch (levels) set to assure details are visible for qualitative presentation. Click here for file
