# Supplementary figures and images for: Stromal matrix metalloprotease-13 knockout alters Collagen I structure at the tumor-host interface and increases lung metastasis of C57BL/6 syngeneic E0771 mammary tumor cells
Source: BMC Cancer. 2013 Sep 5;13:411. doi: 10.1186/1471-2407-13-411 (PMC3766650; doi:10.1186/1471-2407-13-411)

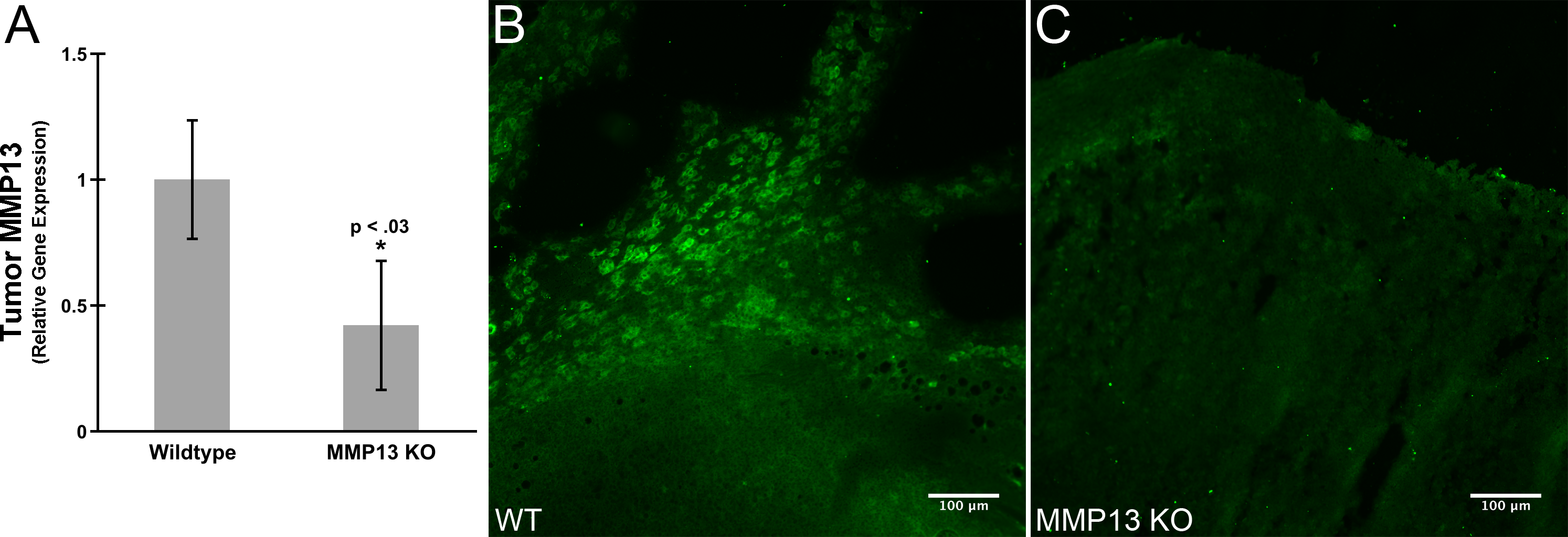

Supplement: Additional file 1: Figure S1 — Decreased MMP13 expression in MMP13 KO tumors. WT and MMP13 KO mice were implanted with E0771 mammary tumors as described. (A) Following excision of the primary tumor, MMP13 gene expression was assessed by quantitative PCR (qPCR) and normalized to 1, showing decreased MMP13 expression in MMP13 KO versus WT tumors (p < .03) in the same cohort of WT (n=6) and MMP13 KO (n=4) mice. In addition, following immunofluorescence labeling for MMP13, the tumors from the (B) WT mice had peritumor MMP13+ cell bodies which were not apparent in the tumors from the (C) MMP13 KO mice. To assure details are visible for illustrative purposes, the original grayscale MMP13 immunofluorescence is shown with “Green” LUT applied in ImageJ, with levels (screen stretch) linear and set the same for both images. [file 1471-2407-13-411-S1.tiff]
